# In Vivo Validation of Computational Fluid Dynamics for Determining the Pressure Gradient for Multi-segmental Femoropopliteal Disease

**DOI:** 10.1007/s00270-026-04427-1

**Published:** 2026-04-26

**Authors:** L. van de Velde, L. Rutten, M. van Werkum, P. Cernohorsky, E. Groot Jebbink, M. Versluis, M. M. P. J. Reijnen

**Affiliations:** 1 Multi-Modality Medical Imaging Group (M3I), TechMed Centre, University of Twente, Enschede, The Netherlands; 2 Department of Surgery, Rijnstate, Rijnstate Hospital, Wagnerlaan 55, Mailbox 5500, 6800 TA Arnhem, The Netherlands; 3 Physics of Fluids Group, TechMed Centre, University of Twente, Enschede, The Netherlands; 4 Department of Radiology, Rijnstate, Arnhem, The Netherlands; 5 Department of Surgery, Amsterdam UMC location University of Amsterdam, Meibergdreef 9, Amsterdam, The Netherlands

**Keywords:** Peripheral artery disease, Femoral artery, Pressure gradient, Computational fluid dynamics, Fractional flow reserve

## Abstract

**Purpose:**

In peripheral arterial disease, the presence of multi-segmental (adjacent) stenotic lesions is common. The application of anatomic grading and duplex ultrasound for defining the significance of multi-segmental lesions is limited. This study aimed to validate computational fluid dynamics (CFD) simulations for estimating the pressure gradient in patients with multi-segmental femoropopliteal disease.

**Materials and Methods:**

Fifteen patients scheduled for angiography with multiple lesions in the femoropopliteal artery were prospectively enrolled. Pressures were recorded in rest and after papaverine administration using sheath and pressure-wire measurements. The resting pressure gradient of the full segment and the fractional flow reserve (FFR) of the full segment and individual lesions were calculated. These metrics were noninvasively replicated by CTA-based CFD using a reference flow rate (CFDm) and a duplex ultrasound-derived flow rate (CFDi). The agreement and diagnostic accuracy of CFD for identifying hemodynamic significance was assessed.

**Results:**

CFDm underestimated the pressure gradient by − 4.0 ± 6.2 mmHg (mean ± SD). CFDi closely approximated the measured gradient with a 0.1 ± 3.1 mmHg difference. The full-segment FFR had a − 0.02 ± 0.06 difference and the individual-lesion FFR had a 0.01 ± 0.06 difference relative to the measured FFR. The diagnostic accuracy of CFD for the resting pressure gradient was 73% (CFDi) and 80% (CFDm). CFD demonstrated a diagnostic accuracy of 89% for full-vessel FFR and 83% for individual-lesion FFR.

**Conclusion:**

The results provide proof-of-concept validation for the use of CFD for noninvasive assessment of multi-segmental femoropopliteal disease. This could guide the treatment plan of multi-segmental PAD. *Level of Evidence*: Level 3b, Individual case–control study.

**Graphical Abstract:**

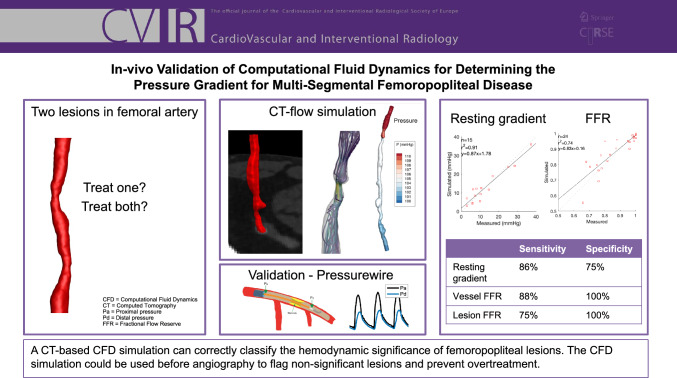

**Supplementary Information:**

The online version contains supplementary material available at 10.1007/s00270-026-04427-1.

## Introduction

Patients with peripheral arterial disease (PAD) commonly present with multilevel (e.g., iliac and femoral) or multi-segmental (two stenoses in one-vessel segment) disease. During angiography, multi-segmental disease is present in about 41% of femoropopliteal arteries [1]. It can be difficult to determine which of the multi-segment lesions warrant treatment. As any additional treatment induces the risk of complications, targeted treatment is key.

The severity of peripheral lesions is usually quantified by CTA and/or by measuring peak systolic velocity ratios (PSVR) using duplex ultrasonography (DUS) [2]. PSVR has a reasonable correspondence with pressure gradients over a single stenosis [3], but its value for multi-segmental disease is limited [4]. No adequate reference velocity for PSVR is present [1] and hemodynamic interactions between stenosis [5] are not accounted for. Ideally, in these cases the pressure gradient is measured [6, 7]. However, pressure measurements are invasive and the materials required are often unavailable. It would therefore be beneficial to determine the pressure loss noninvasively before angiography.

Computational fluid dynamics (CFD) can estimate pressure gradients using imaging data and by setting appropriate boundary conditions. For PAD, previous studies have validated a pressure assessment for iliac artery stenosis [8, 9]. This study aimed to validate the CFD-predicted pressure gradient in patients with multiple femoropopliteal lesions, using CTA and DUS as input, against pressure-wire measurements.

## Methods

### Patient Study

This study was designed as a prospective single-center study. The study was approved by the regional Medical Ethics Committee (CMO-2016–2431), registered in the Netherlands Trial Register (NL9744), and conducted in accordance with the Declaration of Helsinki.

### Study Population

No sample size calculation was performed for this feasibility study. Fifteen patients with multiple stenotic lesions (> 50%) in the superficial femoral artery (SFA) and/or popliteal artery (PA) scheduled for endovascular treatment were included. Baseline patient factors were reported according to PAD guidelines [10]. The Supplemental Material provide further inclusion and exclusion criteria [11].

### Invasive Pressure Measurements

The pressures proximal and distal to the multi-segment lesions were measured on the sheath and a 0.014″ pressurewire (PressureWire X, Abbott, Abbott Park, IL) in rest and after vasodilator administration (30 mg papaverine). From the proximal (Pa) and distal pressure (Pd), the resting pressure gradient and the peripheral fractional flow reserve were calculated (Table 1). Additional details are given in the Supplemental Material.

**Table 1:** Analytical parameters

*Pressure signals*	
Proximal pressure	$P_{a}$
Distal pressure	$P_{d}$
Resting pressure gradient	$P_{a} - P_{d}$
Peripheral fractional flow reserve (FFR)	$\frac{{P_{d} }}{{P_{a} }}$, during vasodilation
*CFD conditions*	
DUS spatial peak velocity	$\overline{v}_{p}$
DUS cross-sectional area	$A$
DUS flow rate	$Q = \frac{{\overline{v}_{p} }}{2}A$
Blood serum hematocrit	$\eta$
Blood viscosity	$1.2 \cdot \left( {1 + 2.5\eta + 6.2\eta^{2} } \right)$

*CFD* Computational Fluid Dynamics, *DUS* Duplex ultrasound

### Computational Fluid Dynamics Simulations

The equations of blood flow were solved with CFD simulations using SimVascular (Version 2022.07.20 [11]), which has been validated for stenotic flows in the femoral artery [12]. A rigid wall and constant viscosity model based on the patient’s hematocrit were used (Table 1, [13]). The CFD model employed the patient’s anatomy segmented from CTA using a level-set method [13].

Two strategies were evaluated for setting the resting inflow rate: a uniform resting flow rate of 1.26 mL/s (CFDm) and a DUS measurement in the PA (CFDi). DUS measurements (Hitachi L35 probe, Fujifilm, Tokyo, Japan) obtained in the PA were used. As a systematic overestimation of peak velocity for Hitachi systems by 8–19% has been reported [14], a correction of the measured velocity of 0%, − 10% and − 20% was applied. Using an in-house MATLAB script (version 2024b, MathWorks, Natick, MA), velocity tracings over time were extracted to compute the DUS flow rate used for CFDi (Table 1). For hyperemia, the DUS-based simulation was used with a peripheral resistance reduced by a factor of 3.

More details on the CFD mesh and boundary conditions are provided in the Supplemental Material.

### Statistical Analysis

Data are presented as mean ± standard deviation. The simulated values were compared to the measurements with a Bland–Altman analysis [15]. Student’s t test of the differences was used to test if the mean difference was statistically different than zero (significance level = 0.05). The limits of agreement were calculated as the mean difference ± 1.96 times the standard deviation. To assess diagnostic accuracy, we analyzed if CFD correctly classified the stenotic lesions as significant compared to the measurements and calculated the accuracy, sensitivity, and specificity, with confidence intervals by the Clopper–Pearson intervals. Multi-segmental lesions were considered significant if the resting gradient exceeded 10-mmHg, and for FFR < 0.8[6] (both for multi-segmental and individual FFR).

## Results

An overview of the patient population is provided in Table 2. For the 15 patients, an average resting pressure gradient of 12.8 ± 10.1 mmHg was measured over the multi-segmental lesions.

**Table 2:** Patient characteristics (*N* = 15)

	All patients (*N* = 15)	Hyperemic measurement (*N* = 9)
Age	70 ± 9	68 ± 8
Race	100% Caucasian	100% Caucasian
Sex	73% male (*N* = 11)	89% male (*N* = 8)
Number of stenoses	3 ± 1.5	3 ± 1.2
Rutherford category		
1	1	0
2	2	1
3	6	6
4	2	0
5	3	2
6	1	1
Diabetes mellitus		
Not requiring insulin	3	2
Requiring insulin	3	3
Type 1	2	2
Hypertension	93% (*N* = 14)	100% (*N* = 9)
Smoking	4 active, 10 past (> 6 months), 1 never	2 active, 6 past, 1 never
Renal function (eGFR, CKD-EPI, mL/min/1.73m^2^)	66 ± 24	66 ± 22
Hyperlipidemia	93% (*N* = 14)	100% (*N* = 9)
Cardiac status	10 asymptomatic, 5 past MI	5 asymptomatic, 4 past MI
Patent below-the-knee arteries		
* N* = 0	1	1
* N* = 1	2	1
* N* = 2	5	2
* N* = 3	7	5

*eGFR* estimated glomerular filtration rate, *CKD-EPI* Chronic Kidney Disease Epidemiology Collaboration, *MI* myocardial infarction

Measurement of FFR was performed in nine patients. Papaverine was not administered due to a QTc-time exceeding the threshold (*N* = 5) or hypomagnesemia (*N* = 1). The mean measured FFR across the multi-segment lesions was 0.67 ± 0.16. In this subcohort, 24 distinct lesions were present with an individual-lesion FFR of 0.80 ± 0.11. Sixteen lesions received treatment, of which 10/16 had an individual FFR < 0.8.

**Fig. 1:**
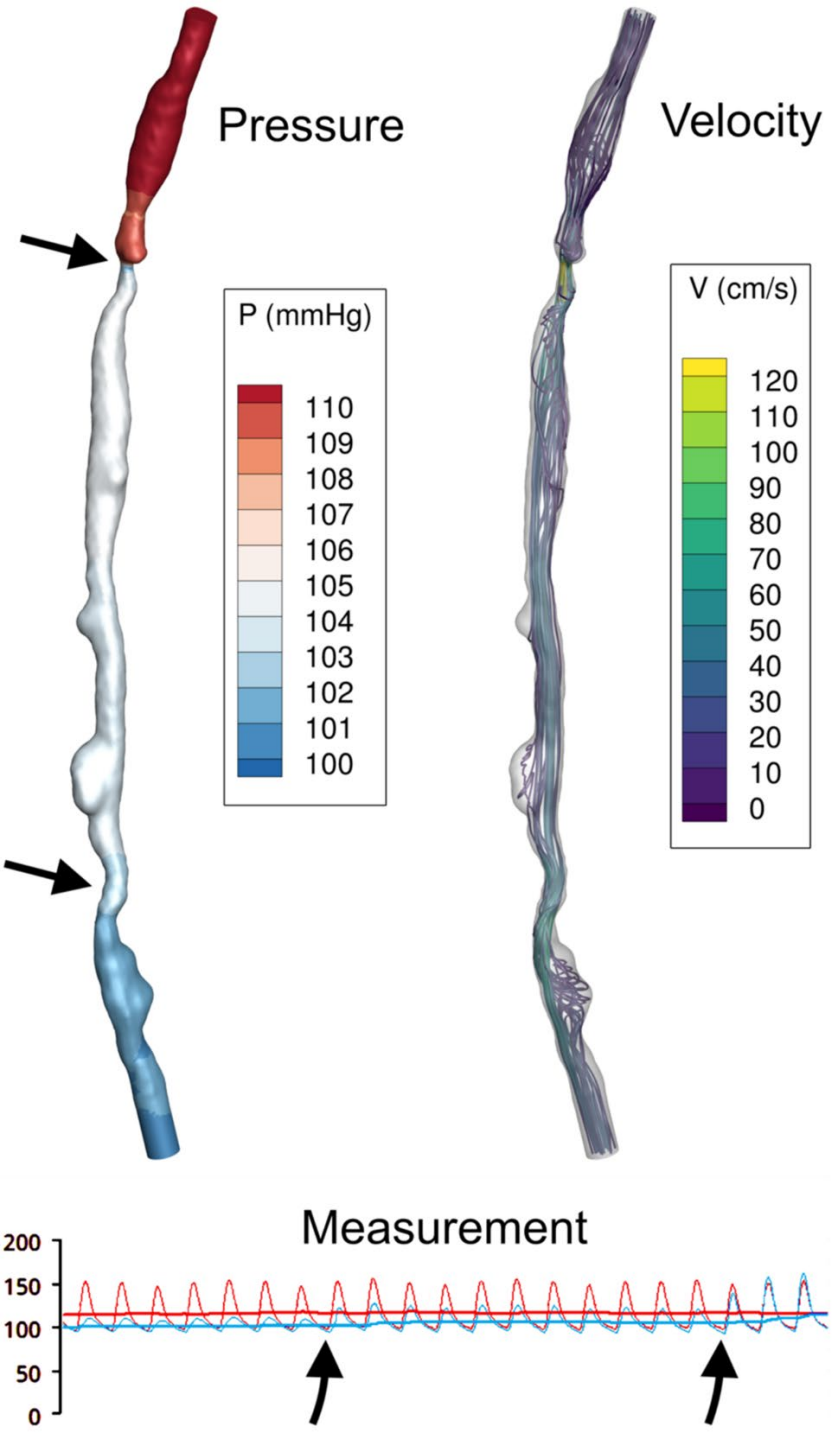
Simulated hemodynamics (top figures) and measured pressure (down) for a case. The simulated mean aortic pressure across the superficial femoral artery by CFD is shown top left. Two lesions with a drop in pressure are indicated by arrows. The top right figure shows the associated velocity streamlines. The pullback measurements of the pressure wire (blue) and proximal pressure (red) are shown in the lower plot, with the pressure curve data as well as the mean aortic pressure (straight line) visible. The pressure-wire pullback shows two points with a jump in pressure (arrows), in agreement with the simulated pressure

**Fig. 2:**
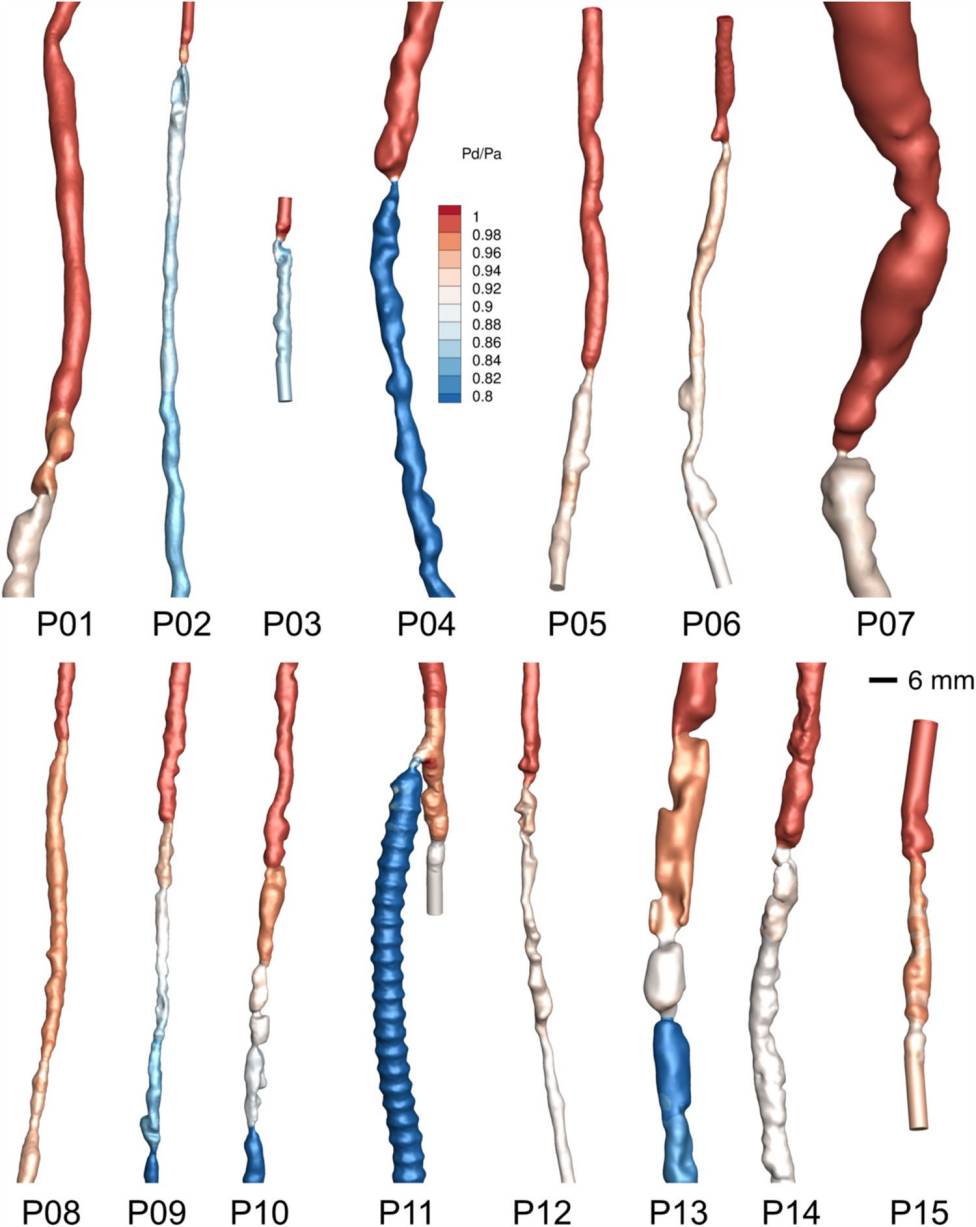
Simulated cases with CFD for the resting condition, based on the flow rate information from duplex ultrasound. The pressure was normalized relative to inlet (most proximal) pressure to allow a representation of the pressure gradients of all cases. P03 was an in-stent restenosis in a covered stent, P11 was a stenosis near the proximal anastomosis of a synthetic bypass graft

Figure 1 illustrates the measured and simulated pressures in a case with diffuse femoropopliteal disease. All geometries and DUS-based simulations in resting condition are displayed in Fig. 2.

**Fig. 3:**
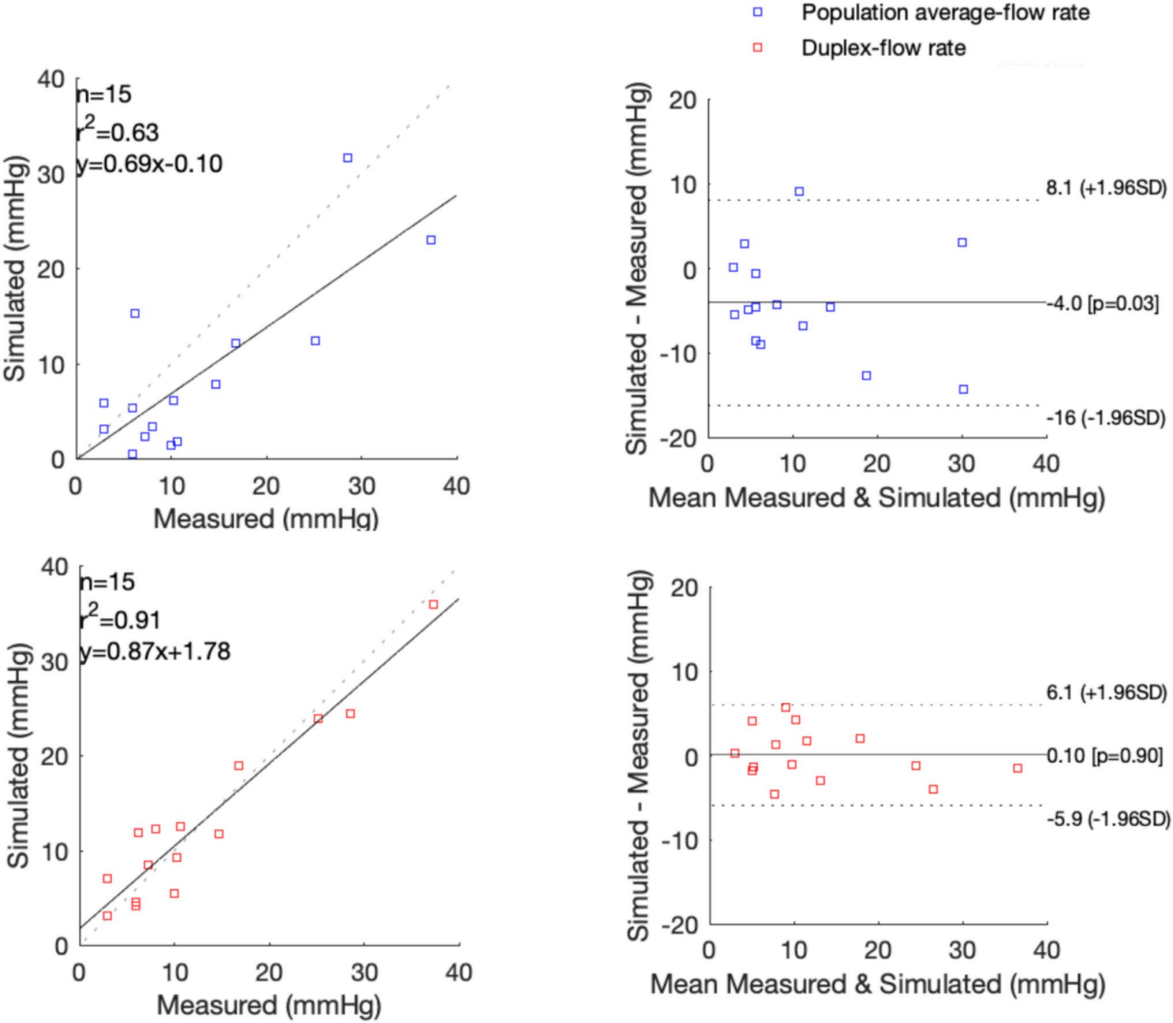
Left: correlation plots between the measured and simulated resting pressure gradient. Right: Bland–Altman plot for the measured and simulated resting pressure gradient. The mean bias (straight line) and the 95%-limits of agreement (dashed lines) are shown. Simulation flow rates were based on a single population average value (blue) or an individualized value measured by duplex ultrasound (red). *r* = Pearson’s correlation coefficient

Table 3 presents the measured and simulated pressure gradients in rest for CFDm and CFDi. CFDm yielded a difference of − 4.0 ± 6.2 mmHg (*p* = 0.03), i.e., an underestimation of the pressure gradient. CFDi was optimal when a − 10% peak velocity correction was applied with a 0.1 ± 3.1 mmHg difference (p = 0.90; no correction 2.8 ± 4.0 mmHg and -20% correction − 2.1 ± 3.5 mmHg). The Bland–Altman analysis (Fig. 3) showed no relation between the pressure gradient and the simulation error.

**Table 3:** Results resting flow

Subject	Measurement	Simulation PA		Simulation DUS	
	Pdiff (mmHg)	Flow rate (mL/s)	Pdiff (mmHg)	Flow rate (mL/s)	Pdiff (mmHg)
1	5.9	1.26	5.3	2.90	4.6
2	28.5		31.6	1.08	24.5
3	8.0		3.4	2.64	12.3
4	25.1		12.4	1.81	23.9
5	3.0		3.1	2.08	7.1
6	10.3		6.1	1.64	9.3
7	10.0		1.4	2.08	5.5
8	2.9		5.9	0.92	4.1
9	14.7		7.9	1.60	11.7
10	16.8		12.2	1.60	18.9
11	7.2		2.4	3.26	8.7
12	6.2		15.3	1.08	11.9
13	37.3		23.0	2.18	36.9
14	10.7		1.8	3.78	12.4
15	5.9		0.5	4.2	5.0

*PA* population average flow rate, equal for all patients

*DUS* duplex ultrasound flow rate, with 10% peak systolic velocity correction

*Pdiff* pressure gradient over the full-vessel segment

**Fig. 4:**
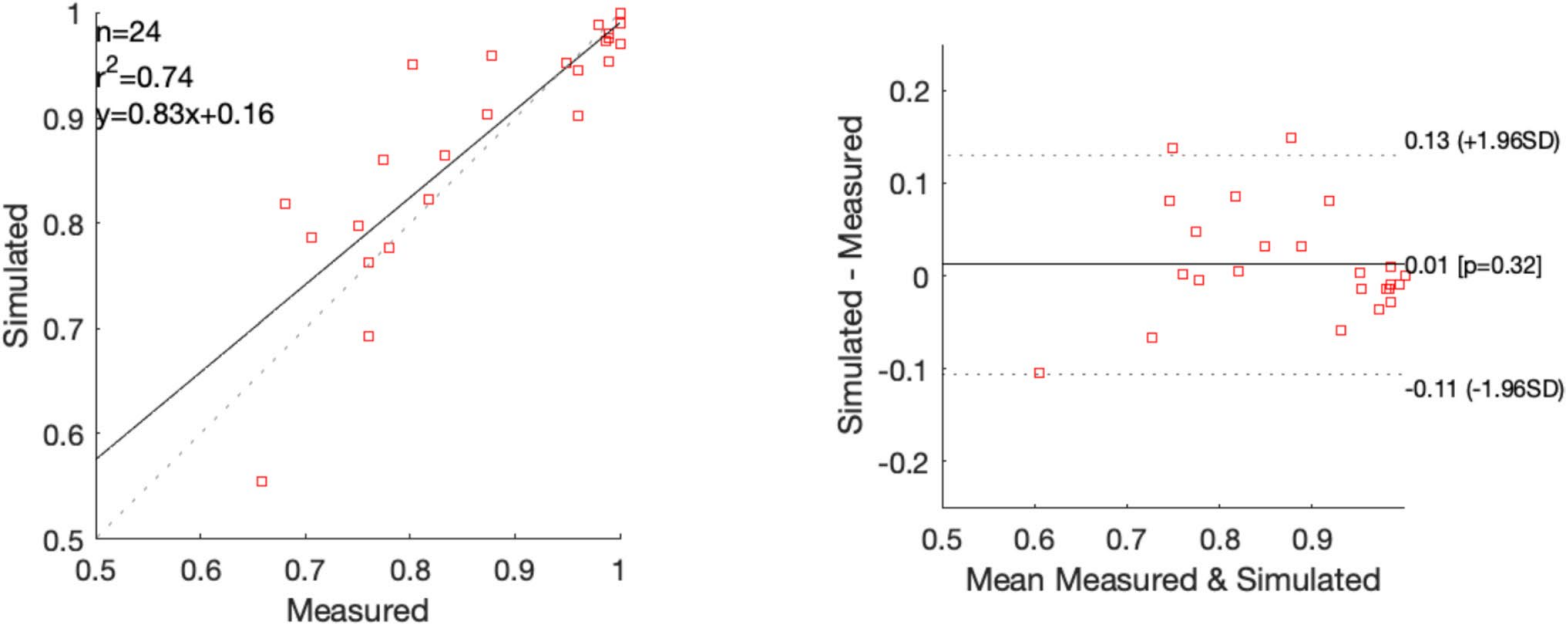
Left: correlation plot between the measured and simulated fractional flow reserve (FFR) of individual lesions. Right: Bland–Altman plot of the differences between the measured and simulated FFR. The mean bias (straight line) and the 95%-limits of agreement (dashed lines) are shown. *r* = Pearson’s correlation coefficient

For the hyperemic condition, the full-segment FFR is presented in Table 4. CFD demonstrated a difference in FFR of − 0.02 ± 0.06 relative to the measured FFR. For the individual-lesion FFR, CFD provided a 0.01 ± 0.06 difference compared to the pullback measurements (Fig. 4).

**Table 4:** Fractional flow reserve

Subject	Measurement		Simulation	
	Pd/Pa resting	FFR	FFR	Flow rate (mL/s)
1	0.93	0.69	0.73	6.9
2	0.62	0.41	0.33	1.4
5	0.97	0.79	0.75	4.4
6	0.91	0.67	0.70	3.3
7	0.89	0.68	0.81	4.7
8	0.97	0.97	0.91	1.2
10	0.78	0.51	0.57	2.1
12	0.94	0.63	0.67	2.1
15	0.92	0.72	0.73	11.5

*Pd/Pa* ratio of distal pressure to the proximal pressure of the full-vessel segment in resting conditions, *FFR* fractional flow reserve over the full-vessel segment, equal to the ratio of the distal pressure to the proximal pressure in the vasodilator condition

### Diagnostic Accuracy

For resting gradients, CFD demonstrated a sensitivity of 57% [95%-CI 18%–90%] and 86% [42%–100%], a specificity of 88% [47%–100%] and 75% [35%–97%], and a diagnostic accuracy of 73% [45%–92%] and 80% [52%–96%] for CFDm and CFDi, respectively. For the full-segment FFR, CFD had a sensitivity of 88% [47%–100%], a specificity of 100% [2.5%–100%] and a diagnostic accuracy of 89% [52%–99%]. For the individual-lesion FFR, CFD had a sensitivity of 75% [35%–97%], a specificity of 100% [79%–100%] and a diagnostic accuracy of 92% [73%–99%].

## Discussion

This study investigated the accuracy of CFD for assessing the pressure gradient in patients with multiple femoropopliteal lesions. The DUS-based simulation showed high accuracy in resting and hyperemic (exercise) conditions, although the latter was validated only in a subset of patients. The results provide an initial proof-of-concept validation of the CFD model for multi-segmental femoropopliteal disease, requiring confirmation in a larger cohort.

The strategy for the flow boundary condition is a key differentiator in CFD simulations. Scaling of the flow rate with the supplied organ volume is effective in some territories [16], but for the femoropopliteal artery we chose to use a direct measurement using DUS. Although challenged by a 20% interobserver variability of DUS [17], this approach demonstrated excellent results. For our Hitachi/Fujifilm system we found that a 10% correction was required, which might be machine-dependent [14].

For the FFR, an excellent specificity was demonstrated for the CFD assessment. [5] The CFD method is therefore well-suited for preventing overtreatment. CFD could be applied for multi-segmental cases that include moderate lesions to make an informed treatment plan of which lesions to treat. An additional advantage of CFD is virtual treatment [18] to assess if the treatment plan will sufficiently resolve the overall pressure gradient.

This study did not investigate treatment guidance for PAD by the pressure gradient. Of the lesions assessed with FFR, 16 were treated, of which only 10 were deemed significant (FFR < 0.8), although this number could increase after treatment of adjacent lesions. These results suggest that current treatment guidance leads to overtreatment, with associated iatrogenic risks of dissection or thrombosis. This stresses the need for trials that investigate if pressure-based (CFD or invasive) treatment guidance improves outcomes.

### Limitations

The uncertainty of pressure gradient estimation is driven primarily by anatomic and flow rate inaccuracies [19], which in turn were subject to limitations in CTA imaging and DUS [20]. Furthermore, collaterals [21] not included in our model may have caused overestimation of the flow rate through high-grade stenotic lesions. For high-grade lesions, an accurate assessment is clinically less relevant, however. The flow rate estimation by DUS could be further improved by using a (larger) sampling volume that encompasses the full vessel or novel ultrasound vector flow imaging techniques [22, 20].

## Conclusions

The study showed excellent agreement and high diagnostic accuracy of CFD simulations, using CTA and DUS as input, for an assessment of the hemodynamical significance of multi-segmental femoropopliteal disease.

## Supplementary Information

Below is the link to the electronic supplementary material.

Supplementary file1 (DOCX 51 KB)

Supplementary file2 (DOCX 51 KB)

## Abbreviations

CAD: Coronary artery disease
CFD: Computational fluid dynamics
CFDm: CFD with a general inflow condition
CFDi: CFD with an individualized inflow condition by duplex ultrasound
DUS: Duplex ultrasound
FFR: Fractional flow reserve
PAD: Peripheral arterial disease
PC-MRI: Phase Contract Magnetic Resonance Imaging
SFA: Superficial femoral artery
PA: Popliteal artery
