# Supplementary material for: In Vivo Validation of Computational Fluid Dynamics for Determining the Pressure Gradient for Multi-segmental Femoropopliteal Disease
Source: Cardiovasc Intervent Radiol. 2026 Apr 26;49(6):1190–8. doi: 10.1007/s00270-026-04427-1 (PMC13212610; doi:10.1007/s00270-026-04427-1)
Supplement: Supplementary file 1 — Supplementary file1 (DOCX 51 KB) [file 270_2026_4427_MOESM1_ESM.docx]

## Supplemental material - Expanded Methods

### Study population

Patients were eligible for enrollment if CTA and DUS within the previous 6 months were available. Patients were excluded in case of total occlusion of the SFA or PA, history of atrial fibrillation, antiarrhythmic drug use, drug use with a “Known risk or Possible risk of Torsades-de-Pointes” (https://crediblemeds.org), atrioventricular block, intraventricular conduction delays or hypokalemia. The cardiac exclusion criteria were applied due to the risk of intra-arterial papaverine to induce ventricular arrhythmias [1].

### Invasive pressure measurements

For the procedure, a 5-6 Fr sheath was positioned under ultrasound guidance in the common femoral artery and advanced into the SFA, unless a proximal SFA stenosis was present. A pressure transducer (Medline Namic, Northfield, IL), kept at stable height, was connected to the sheath and continuously flushed with heparinized saline. The 0.014" pressurewire was positioned at the tip of the sheath and was calibrated to the sheath pressure. The stenotic lesions were crossed with a standard wire, or where possible, with the pressure-wire. At the discretion of the interventionalist, a second 0.014" safety-wire was positioned distal to the lesions to prevent repeated crossing of high-grade lesions.

A resting measurement was performed, and, in case of normal QTc (men <450 ms, women <460 ms), a hyperemic pullback measurement was performed after papaverine administration through the sheath. The hyperemic pulback was initiated when a maximal vasodilator response was observed, typically within 15 seconds of papaverine administration. After the measurements, a drift check was performed, and measurements were corrected if the relative drift exceeded a value of 0.02.

For the measurement analysis, the proximal pressure (Pp) and distal pressure (Pd) signals were averaged over three cardiac cycles. For determining the full vessel segment gradient and FFR, encompassing all stenotic lesions, Pp and Pa before the pullback were used. Individual lesion FFR was assessed as the hyperemic Pd/Pp ratio proximal and distal to lesions quantified as >50% on subtraction angiography.

### Computational fluid dynamic simulations

[2]

*Computational mesh convergence*

A three-element prismatic boundary layer was applied for the meshing of the geometries, 1000 timesteps of 0.1 ms were simulated and the meshes were considered converged if the inlet pressure averaged at three timepoints (0.06, 0.08 and 0.1 s) did not change > 1% for a doubling of the mesh size.

*Boundary conditions and duplex-ultrasound measurements*

Two strategies were evaluated for setting a parabolic steady inlet flow rate boundary condition. The first used a uniform resting flow rate of 1.26 mL/s for all patients, based on previously reported phase-contrast MRI flow rate measurements in the PA (1.4±0.5 [2] and 0.9±0.6 mL/s [3] in 10 and 5 patients with Rutherford 1-3, respectively).

Duplex ultrasound recordings in the popliteal artery were performed by experienced vascular sonographers according to clinical guidelines (sampling volume 1.5-2.5 mm, Doppler angle equal or below 60 degrees [4]).

For the outlet boundary condition, a resistance value was tuned such that inlet pressure equaled the mean arterial arm cuff pressure. For two cases with an origin stenosis in the superficial femoral artery (SFA), the common (CFA) and deep femoral artery (DFA) were included in the models, for which a CFA flow rate twice the targeted SFA flow rate was set and a resistive boundary condition for the DFA was tuned to achieve the target flow rate in the SFA.

*Hyperemic boundary condition*

For the simulation of maximal flow during hyperemia, the DUS-based simulation was used as starting point and the distal (SFA) resistance was decreased by a factor of 3 to simulate vasodilation of the microvasculature [5]. As previous PC-MRI studies were unable to reliably assess the increase in mean flow rate during hyperemia [2], this factor was empirically chosen.

#### References

1. Nakayama M, Tanaka N, Sakoda K, Hokama Y, Hoshino K, Kimura Y, et al. Papaverine-induced polymorphic ventricular tachycardia during coronary flow reserve study of patients with moderate coronary artery disease: Analysis of ECG data. Circulation Journal. 2015;79:530–6. https://doi.org/10.1253/circj.CJ-14-1118

2. van de Velde L, Groot Jebbink E, Hagmeijer R, Versluis M, Reijnen MMPJ. Computational Fluid Dynamics for the Prediction of Endograft Thrombosis in the Superficial Femoral Artery. Journal of Endovascular Therapy. SAGE Publications Inc; 2022;30:615–27. https://doi.org/10.1177/15266028221091890

3. Versluis B, Dremmen MHG, Nelemans PJ, Wildberger JE, Schurink GW, Leiner T, et al. MRI of arterial flow reserve in patients with intermittent claudication: Feasibility and initial experience. PLoS One. 2012;7:e31514. https://doi.org/10.1371/journal.pone.0031514

4. Pena CS, McCauley TR, Price TB, Sumpio B, Gusberg RJ, Gore JC. Quantitative blood flow measurements with cine phase-contrast MR imaging of subjects at rest and after exercise to assess peripheral vascular disease. American Journal of Roentgenology. 1996;167. https://doi.org/10.2214/ajr.167.1.8659362

6. Gerhard-Herman M, Gardin JM, Jaff M, Mohler E, Roman M, Naqvi TZ. Guidelines for noninvasive vascular laboratory testing: A report from the American Society of Echocardiography and the Society for Vascular Medicine and Biology. Vascular Medicine. 2006;11:183–200. https://doi.org/10.1177/1358863x06070516

7. Taylor CA, Fonte TA, Min JK. Computational fluid dynamics applied to cardiac computed tomography for noninvasive quantification of fractional flow reserve: Scientific basis. J Am Coll Cardiol. 2013;61:2233–41. https://doi.org/10.1016/j.jacc.2012.11.083
